# Determinants of fatal car accident risk in Finote Selam town, Northwest Ethiopia

**DOI:** 10.1186/s12889-020-08760-z

**Published:** 2020-05-06

**Authors:** Melaku Tadege

**Affiliations:** Department of Statistics, Injibara University, Injibara, Amhara Ethiopia

**Keywords:** Road traffic accident, Ethiopia, Finote Selam, Amhara, Ordinal logistic regression

## Abstract

**Background:**

In the globe, 1.3 million deaths and around 50 million non-fatal injuries were reported. From all deaths, 90% occur in developing countries. Ethiopia is considered as one of the worst countries in the globe where road traffic accident causes a lot of fatalities and injuries of road users every year. The main objective of the study was to identify the main predictors of fatal car accident.

**Methods:**

The retrospective research design was applied. 255 records were taken from Finote Selam traffic police office, northwest part of Ethiopia from September 2009 to January 2018. The statistical analysis was performed by using SPSS version 23 software. Chi-square for association test and ordinal logistic regression for predictor identification were used.

**Results:**

Age of drivers were the responsible causes of fatal road traffic accident (*p*-value = 0.033). The more experienced drivers decreased the occurrence of fatal traffic accidents. In addition, increasing vehicle service year reduced the occurrence of accidental death. Besides, the occurrence of fatal car accident in autumn season was 0.44 times less than that of in summer season. Additionally, drivers’ educational level was played a crucial role in a road traffic accident. For instance, drivers whose educational level was below 12th grade were the most responsible factor for car accident deaths. What is more, it was seen that drivers who drove their vehicles could minimize the occurrence of fatal traffic accident (*p*-value = 0.010).

**Conclusion:**

In conclusion, fatal road traffic accidents happened due to drivers’ lack of sufficient driving experience and low educational level. In addition, driving on weekends and driving on summer season were disclosed as responsible for fatal car accident. Moreover, drivers with younger age and those who drove a new vehicle likely caused fatal car accident. However, drivers who drive their vehicles seemed to be less responsible for fatal car accident than that of employed.

## Background

A road traffic accident can be stated as a collision among vehicles, between pedestrians and vehicles, between vehicles and animals, or between fixed obstacles and vehicles. This collision leads to human fatal injury [1]. In the globe, 1.3 million deaths and around 50 million serious and slight damages were reported [2]. It is one of the top two causes of death among the most economically active population age group 15–44 years old [3]. Furthermore, above 75% of road traffic accident fatalities occur in this age group [4–6]. The burden of road traffic accident is disproportionally huge in LMICs [5, 7, 8]. Fatal RTAs are at least two-times common in LMICs than high-income countries. Africa covers the highest annual rate of road fatalities in the world 27 per 100,000 people [9]. In the future, the difficulty can even rise because of accelerated economic growth and an increase in the number of vehicles in the continent [4, 7, 10]. Despite the growing burden of road traffic accidents, road safety remains a forgotten issue in LICs and the health sector has been slow to acknowledge it as a priority public health problem [9, 11]. From all deaths, more than 90% occur in LICs. 70% of the deaths involve vulnerable road users, with 35% of pedestrian deaths being children [12].

As the number of vehicles in developing countries continues to rise, the road traffic problem will increase. It is estimated that road traffic cost in LMICs is more than $100 billion every year, or it is 1–3% of their gross national product because of disability, premature death, loss of productivity, medical expenses, and material damages [13]. The road traffic costs and related injuries were not prioritized in LMICs [14]. According to world health organization report, 90% of the world’s fatalities on the roads occurred in LMICs even though LMICs have below 50% of the world’s registered vehicles [12]. By 2020 RTAs are estimated to be the third main cause of deaths in the world [7].

The burden of RTAs is too much greater in Africa continent than anywhere else due to many exposed road users are involved, overcrowding, poor transport conditions such as lack of seat belts, and hazardous vehicle environments. Under-reporting system has also hidden the consequence of the problem in Africa [15]. Most countries in Africa have insufficient policies and strategies to protect exposed road users. Moreover, measurements on predictors like alcohol-impaired driving, speed control, seat belt, child restraints, and helmet use are not common in most countries of this continent. In addition, there is limited traffic law enforcement traffic regulation in the Africa region [13]. In the LMICs from Africa countries, the annual RTAs rate is 32.3 fatalities per 100,000 population, while America has around 16 fatalities per 100,000, Europe continent has 13.4 fatalities per 100,000 and South East Asia has also 16.6 fatalities per 100,000 population [13]. Road traffic accidents constitute major economic, developmental challenges of developing countries, and health especially adversely hit sub-Saharan African countries [16]. Around 90% of the traffic crashes occurred in LMICs of which Sub-Saharan countries had faced the highest fatality rate of 28.3 per 100,000 people, which is substantially higher than any continent in the world [17].

A study in Italy stated that male drivers were the main responsible factor for fatal accidents as compared to female drivers [18]. Another study in Seoul city discussed female drivers were correlated with the level of accident severity [19]. Drivers who are less educated were more likely to be involved in a fatal traffic accident [20–22]. Younger age drivers were one of the significant predictors of fatal traffic accidents [19, 23, 24]. The risk factors of a road traffic accident were daylight and driver’s age [25]. The risk of drivers involved in road traffic accidents decreased from 15.9% in the first year of getting a driving license to 3.13% after 10 years’ of driving experience [26]. Drivers who drive on the weekend and at night have been correlated with severe casualties [22]. A study conducted based on logistic regression analysis showed that injury severity is the most commonly sustained within the vehicle type [27]. An investigation in Iran concludes that the majority of fatal accidents happened during the summer season [28].

Ethiopia is considered as one of the worst countries in the globe where RTAs cause a lot of fatalities and injuries of road users every year [12] and has the highest rate of RTAs because road transport is the main transportation system in the country. The accident has been worsened as the number of vehicles has increased consequently due to increased traffic flow and collision between vehicles and pedestrians. RTA is one of the significant obstacles in the road transport sector in the country. Every year, many people are lost their lives and the huge property is damaged due to RTAs in the country. Ethiopia loses around 400 million Ethiopian birr each year because of RTAs and the third killing vector [29]. Like other African countries, Ethiopia is facing a huge road safety crisis. Annually, thousands of people are killed and most of them are from economically active population age group. According to WHO, in 2013 the prevalence of RTAs in Ethiopia was 25.3 per 100,000 population which is one of the highest rates in the world [30]. During 2007/8, 15,082 road traffic accidents happened in Ethiopia. Of them, 2161 were killed while 7140 were slight and serious injuries [31].

The occurrence of RTAs is rising in the country as the vulnerability to this risk increases with rapid population growth, rapid motorization, and increase in the road network coupled with a poor attitude and safety habits of road users [32]. There is no well-defined road safety policy, strategy, or program in the country. Records stated that about 12,140 death and 29,454 injuries happened between 2005 and 2011 in Ethiopia [33]. Amhara region accounted for 27.3% of the entire RTA related fatalities in Ethiopia during the year 2008/9, which is the highest figure among all regions [34]. Finote Selam town is one of the administrative zones of Amhara region. According to the traffic police office report in the Finote Selam town, the prevalence of the occurrence of fatal car accident is dramatically increasing from time to time. As a result, the main aim of this investigation was to identify the main responsible predictors of fatal car accident in the Finote Selam town.

## Methods

### Study area

Finote Selam is a town located in Amhara regional state, 387-km far from Addis Ababa and 176-km far from Bahir Dar. The air travel, the shortest distance between Finote Selam and Addis Ababa is 246-km. Finote Selam, the “Pacific Road”, the name given by Emperor Haile Silassie during the Italian attack on Ethiopia. Formerly its name was Wojet. Now Finote Selam is the capital city of West Gojjam Zone. It is surrounded by Jabi Tehna Woreda. According to the 2007 national census conducted by the central statistical agency of Ethiopia, Finote Selam town has a total population of 25,913. From all population, 95.91% were Ethiopian Orthodox Christianity followers, while 3.34% were Muslim.

### Study design

The study used a retrospective study design [35]. All records from September 2009 up to January 2018 were considered in the study. Secondary data from the Finote Selam traffic police office records were taken. There were 312 road traffic accidents in the given time interval. Of which, a total of 255 records were included in the study in the given time interval.

### Ordinal logistic regression

There are many occasions when the response variable is multi-level. Outcome variable can be grouped into two categories-multinomial and ordinal. When the outcome variable is classified in a certain order, it is not possible to use the multinomial logistic regression model. In such a case, ordinal logistic regression models have been used to analyze ordinal response variables. Moreover, when there is a need to consider several factors, special multivariate analysis for ordinal data is the natural alternative. Ordinal logistic regression models have been widely applied in most investigation. The commonly used ordinal logistic regression model is the constrained cumulative logit model [36].

### The cumulative logit model

Ordinal logistic regression refers to the case where the dependent variable has an order. The most common ordinal logistic model is the proportional odds model, also called cumulative probabilities of the response categories. If we pretend that the dependent variable is recorded as ordinal having categories, then the application of ordinal logistic model is the appropriate method. An attempt to extend the logistic regression model for binary responses to allow for ordinal responses has often involved modeling the cumulative logit.

### Variables

The dependent variable was a road traffic accident (slight injury, serious injury and fatal). A fatal accident can be defined as at least one person died immediately or within 30 days due to RTA. Serious injury can be also defined as at least one person was injured and admitted in the hospital, but no death occurred until 30 days. Slight injury is an injury when at least one person required medical care, but no fatalities or injuries that required hospitalization due to RTA. The predictor variables were drivers’ sex (male, female), experience, educational level (below grade 12, grade 12 and above), age, vehicle service year, vehicle and drivers’ relation (owner, employed), vehicle owner (private, government), light condition (daylight, night), vehicle type (minibus, bajaj, cargo, and pickup), season (summer, winter, autumn, and spring) and day (weekend, weekday). The definition of educational level grade 12 is the final year of secondary school. In Ethiopia season is classified as summer season includes June, July and August which are characterized by heavy rainfalls, autumn covers September, October and November which is the harvest time, winter season includes December, January and February which are characterized by the dry season, spring season covers the rest months March, April and May which are infrequent showers.

### Data collection

The secondary data were taken from Finote Selam traffic police station records using a checklist that was prepared based on the road traffic accident registry format. After data collection, data were checked for completeness. Then, data were cleaned and edited by removing missing values using SPSS version 23 software. During data cleaning and editing stage, records with an incomplete variable of interest were excluded from the study.

## Results

### Socio-demographic data

There were 255 vehicle accidents. Of which 60 (23.5%) were fatal, 103(40.4%) were serious injuries, and 92(36.1%) were slight injuries from 2009 up to 2018. From the total of 255 vehicle accidents, almost all 254(99.6%) of them were responsible by male drivers, only one (0.4%) was female. Of all accidents, 85(33.3%) occurred during summer season followed by winter season 61(23.9%). In addition, 180(70.6%) accidents were registered on the weekdays, and the remaining 29.4% were registered on the weekends. Besides, on the subject of drivers’ educational level, the majority of driver’s educational level was below grade 12. Also, more than one-third of vehicles (44.3%) were minibus followed by cargo. Moreover, above 80% of vehicles were private vehicles. Furthermore, the average age of drivers was around 28 years. Similarly, the average of drivers experience was 4.3 years. The vehicle service year on average was 3.9 years. What is more, on the aspect of expected money loss, there were approximately 149,138 USD or 3,430,180 Ethiopian birr lost. 221(86.7%) of car accidents happened in the daylight whereas 34(13.3%) RTAs happened at night (Table 1).

**Table 1:** Descriptive statistics of the outcomes and predictors of car accident in terms of frequency and percent

Variable	category	Frequency(n)	Percent (%)
Outcome/traffic accident type	Slight injury	92	36.1
	Serious injury	103	40.4
	Fatal	60	23.5
Season	Autumn	55	21.6
	Winter	61	23.9
	Spring	54	21.2
	Summer	85	33.3
Day	Weekend	75	29.4
	Weekday	180	70.6
Sex	Male	254	99.6
	Female	1	0.4
Educational level	Below Grade 12	198	77.6
	Grade 12 and above	57	22.4
Vehicle to driver relation	Own	37	14.5
	Employed	218	85.5
Vehicle type	Minibus	113	44.3
	Bajaj	33	12.9
	Cargo	69	27.1
	Pickup	40	15.7
Vehicle owner	Private	205	80.4
	Government	50	19.6
Light situation	Daylight	221	86.7
	Night	34	13.3

### Chi-square test of association

The proportion of death under summer season 33(38.8%) was greater than all other seasons. There was a significant relationship between seasonal variation and road traffic accident (*p*-value = 0.005). Most of the slight and serious injuries happened on the weekday but the prevalence of death increased on the weekends. There was also a significant relationship between day and human vehicle accident (*p*-value = 0.001). Drivers educational level had also a significant relation with human road traffic accident (*p*-value = 0.025). In addition, vehicle to driver relation (*p*-value = 0.041) and light condition (*p*-value = 0.016) also had significant relationship with human vehicle accident outcome (Table 2).

**Table 2:** Drivers related factors associated with RTAs in Finote Selam town, Northwest Ethiopia from September 2009 to January 2018

variable		Chi-square analysis				
		Human road traffic accident outcome				
		Slight injury	Serious injury	Fatal	Chi-square	*P*-value
Season type	Autumn	22 (40.0%)	27 (49.1%)	6 (10.9%)	18.46	.005*
	Winter	23 (37.7%)	28 (45.9%)	10 (16.4%)		
	Spring	22 (40.7%)	21 (38.9%)	11 (20.4%)		
	Summer	25 (29.4%)	27 (31.8%)	33 (38.8%)		
Day	Weekend	20 (26.7%)	22 (29.3%)	33 (44.0%)	24.75	0.001*
	Weekday	72 (40.0%)	81 (45.0%)	27 (15.0%)		
Driver’s educational level	Below Grade 12	66 (33.3%)	78 (39.4%)	54 (27.3%)	7.34	0.025*
	Grade 12 and above	26 (45.6%)	25 (43.9%)	6 (10.5%)		
Vehicle to driver relation	Own	20 (54.1%)	12 (32.4%)	5 (13.5%)	6.40	.041*
	Employed	72 (33.0%)	91 (41.7%)	55 (25.2%)		
Vehicle type	Minibus	34 (30.1%)	45 (39.8%)	34 (30.1%)	6.92	.328
	Bajaj	12 (36.4%)	16 (48.5%)	51 (5.2%)		
	Cargo	30 (43.5%)	26 (37.7%)	13 (18.8%)		
	Pickup	16 (40.0%)	16 (40.0%)	8 (20.0%)		
Vehicle owner	Private	73 (35.6%)	81 (39.5%)	51 (24.9%)	1.07	.585
	Government	19 (38.0%)	22 (44.0%)	9 (18.0%)		

### Ordinal logistic regression

#### Univariate analysis

From the univariate analysis, when the drivers’ age increased by a single year, the chance of making fatal traffic accident was decreased by 0.94 times and this is also one of the significant predictors for fatal vehicle accidents (*p*-value = 0.001). Increasing single-year driving experience made to reduce death traffic accident. A year increment caused to reduce fatal car accident by 0.89 times (*p*-value = 0.001). Similarly, when the vehicle service increased by a single year, the probability to be involved in death accident was decreased by 0.91 times (*p*-value = 0.008). The fatal car accident in summer season was greater than spring, winter and autumn season. The possibility of the occurrence of death RTA on the weekend was 2.92 times greater than weekday (*p*-value = 0.001). Drivers whose educational level below grade 12 were responsible for the occurrence of fatal car accident by 1.97 times as compared with grade 12 and above (*p*-value = 0.017). Drivers who drive their vehicles were caused to reduce the risk of death accident by 0.43 times as compared with employed (*P*-value = 0.014). The fatal RTAs during daylight were 2.59 times greater as compared to driving at night (Table 3).

**Table 3:** The association of fatal RTAs with predictor variables; Crude odd ratio and adjusted odd ratio (COR, AOR) from the ordinal logistic regression

Predictor variable	Category	Ordinal logistic regression result			
		Univariate analysis		Multivariable analysis	
		COR(95%CI)	*P*-value	AOR(95%Cl)	*P*-value
	Drivers age	0.94 (0.91, 0.97)	0.001*	0.96 (0.93,1.00)	.033*
	Drivers experience	0.89 (0.83, 0.94)	0.001*	0.91 (0.85,0.97)	.005*
	Vehicle service in year	0.91 (0.85,0.98)	0.008*	0.90 (0.83,0.98)	.011*
Season	Autumn	0.40 (0.21, 0.76)	0.005*	0.44 (0.22,0.88)	.019*
	Winter	0.47 (0.25, 0.87)	0.017*	0.81 (0.41,1.59)	.536
	Spring	0.47 (0.25, 0.89)	0.021*	0.68 (0.34,1.34)	.262
	Summer (ref)				
Day	Weekend	2.92 (1.74, 4.88)	0.001*	2.74 (1.58,4.75)	.001*
	Weekday (ref)				
Drivers Educational level	Below grade 12	1.97 (1.13,3.45)	0.017*	1.89 (1.02,3.47)	.042*
	Grade 12 and above (ref)				
Vehicle to driver relation	Own	0.43 (0.22,0.84)	0.014*	0.39 (0.19,0.80)	.010*
	Employed (ref)				
Vehicle type	Minibus	1.63 (0.83,3.19)	.154	1.24 (0.54,2.84)	.608
	Bajaj	1.00 (0.42,2.36)	1.000	0.60(.22,1.65)	.321
	Cargo	0.89 (0.43,1.83)	.742	0.83 (0.34,2.02)	.676
	Pickup (ref)				
Vehicle owner	Private	1.23 (0.69,2.19)	.476	0.89 (0.42,1.89)	.763
	Government (ref)				
Light situation	Daylight	2.59 (1.28,5.23)	0.008*	2.36 (1.09,5.14)	.030*
	Night (ref)				

#### Multivariable analysis

When the age of drivers was increased by a year, the possibilities to be involved in the fatal car accident were decreased by 0.96 times and it is one of the predictors of fatal car accident (*p*-value = 0.033). Single-year increase on the drivers’ experience decreases the chance of the occurrence of fatal traffic accident by 0.91 times (*p*-value = 0.005). Regarding vehicle service year, when the vehicle service year was increased by a single year, the occurrence of accidental death was decreased by 0.90 times (*p*-value = 0.011). The risk of involvement in a fatal accident during autumn was lower as compared to in the summer season (*p*-value = 0.019). Among all deaths, most of them were happened on the weekend compared to weekday (*p*-value = 0.001). Drivers’ educational level had been played a crucial role in a road traffic accident. In other words, drivers whose educational level was below grade 12 were the most responsible factor for fatal car accident. And it was a direct cause to increase death prevalence by 1.89 times (*p*-value = 0.042) as compared to drivers with grade 12 and above educational levels. Drivers who drive their vehicles could minimize the risk of fatal accident (*p*-value = 0.010). Driving on daylight increased human fatal accident by 2.36 times (*p*-value = 0.030) as compared to driving at night (Table 3).

## Discussion

Most of the drivers who involved in all RTAs were males, only 0.4% of all types of RTAs involved female driver. This finding is in line with the findings of several other studies [37–42]. More than 55 % of serious/fatal RTA injuries occurred on the weekend. From the multivariate analysis, the chance of the occurrence of fatal RTA injuries on the weekend was 2.74 times greater as compared with weekday. This study is consistent with other reports [43, 44]. The possible reasons are due to people in Finote Selam town travelling more on the weekend, as people typically visit relatives or friends and move around for social issue at the weekend. Most of fatal RTA injuries happened in the daylight. Several studies stated that fatal RTA injuries mostly happen at night [37, 45]. The discrepancy may arise due to most of injuries/fatalities in the area were related to public transport vehicles. But Public transport vehicles did not functional on the night that is why most accidents were recorded on the daylight. In this study, 55% of fatal RTA injuries occurred in the summer season. Summer is a rainy season in Ethiopia and during rainy time RTAs were likely to increase which was consistent with another study [46]. This may be related to failing to control vehicles during the rainy season. The study concludes that when the age of drivers increases, the occurrence of RTAs decreases which is consistent with many studies across the world [31, 45, 47, 48]. Drivers who drive their vehicles could reduce the occurrence of fatal accidents as compared to employed drivers. Another study shows that owner-drivers caused to minimize fatal injuries. Being an owner decreased the likelihood of fatal RTAs which is in line with another study [49]. Drivers’ with lack of sufficient driving experience caused to increase the chance of the occurrence of fatal accidents as compared to those with long years of driving experiences. This may be because lower driving experience drivers usually involve in driving faster than the recommended limits [50–53]. Another similar study reveals that less experienced drivers were the most significant cause of fatalities than the experienced drivers, as inexperienced drivers may not predict very well what will happen on the roadways [54]. Besides, less-educated drivers were more responsible in fatal pedestrian injuries in the town. There is also another study which is reported as less-educated drivers influence their chance of being involved in fatal road traffic accidents [49]. In addition, the new vehicles were a responsible factor as compared to older vehicles. This may be due to the relationship between vehicles and drivers when the vehicle is new, the driver may not know the characteristics of the vehicle easily since it takes time. On the other hand, one of the limitations of the study might be lack of sufficient data related to several important predictors which may affect the severity of RTA, such as socioeconomic status, medical illness, marital status, alcohol use, speed and vehicle defects were not recorded by the traffic police and hence could not be assessed in the study. Another limitation was under-reporting. As a result, the number of records in the study wasn’t adequate. This may affect the conclusion of the study.

## Conclusions

In conclusion, fatal car accident was happened due to drivers’ lack of sufficient experience, drivers with younger age, drivers’ insufficient educational level, drivers who drive a new vehicle, driving on the weekend, vehicle to driver’s relation, and driving on summer season. Special attention should be needed for drivers who drive a new vehicle, less educated drivers, younger drivers, less experienced drivers, driving during the summer season, employed drivers and driving on the weekend. The possible solution might be traffic police should assign on the weekend and use new technology to control the activities of drivers and vehicles. In addition, the government rules towards driver’s license and age limit shall be changed, and comprehensive examination for drivers annually is recommended. At last, investigation on the cause of car accident from several places in the country level is highly appreciated.

## Data Availability

If needed the raw data in excel format for this article is available.

## Abbreviations

WHO: World Health Organization
RTAs: Road traffic accidents
LICs: Low income countries
LMICs: Low and middle income countries
SPSS: Statistical Package for the Social Science
